# Designing and validation of a reproductive health need assessment tool for women experienced domestic violence

**DOI:** 10.1186/s12978-022-01342-9

**Published:** 2022-01-29

**Authors:** Mahrokh Dolatian, Abbas Ebadi, Seyedeh Batool Hasanpoor-Azghady, Anvar Sadat NayebiNia

**Affiliations:** 1 Midwifery and reproductive health research center, Department of midwifery & Reproductive Health, School of Nursing & Midwifery, Shahid Beheshti University of Medical Sciences, Tehran, Iran; 2 Behavioral Sciences Research Center, Life Style Institute, Faculty of Nursing, Baqiyatallah University of Medical Sciences, Tehran, Iran; 3 Department of Midwifery and Reproductive, Nursing Care Research Center (NCRC), School of Nursing and Midwifery, Iran University of Medical Sciences, Tehran, Iran; 4 Department of Midwifery, Karaj Branch, Islamic Azad University, Karaj, Iran; 5 Clinical Cares and Health Promotion Research Center, Karaj Branch, Islamic Azad University, Karaj, Iran

**Keywords:** Violence, Reproductive, Health, Tool, Mixed-method

## Abstract

**Background:**

Violence as a serious health problem and one of the main manifestations of gender inequality brings about adverse health effects for women. Therefore, it is of utmost importance to recognize the reproductive health status of women subjected to violence in order to provide the health services they need. Considering that one of the ways to determine reproductive health status is the use of valid questionnaires in this field, this study aimed to determine the components of reproductive health in domestic violated women and design a valid and reliable assessment tool.

**Methods:**

The present study was conducted based on a mixed-method design. The first part of the study (qualitative section) was conducted based on conventional content analysis. In this part, unstructured in-depth interviews were conducted with 18 violated women and 9 experts. In the next stage, the item pool was formed and the Reproductive Health Needs of Violated Women Scale was designed based on the review of the literature and the results of the qualitative section with 39 items using the Waltz approach. For psychometric assessment of the above instrument, face and content validity, item analysis, and construct validity were examined using exploratory factor analysis.

**Results:**

Based on the results of factor analysis, the four following factors were extracted with a total variance of 47.62: "men's participation", "self-care", "support and health services", as well as "sexual and marital relationships. The internal consistency of the instrument was calculated at α = 0.70–0.89 and α = 0.94 for different constructs and the whole instrument, respectively. Moreover, intra-cluster correlation coefficients were obtained at ICC = 0.96–0.99 and ICC = 0.98 for constructs and the whole instruments, respectively.

**Conclusions:**

Based on the results of the current study, the present scale is a tool that specifically assesses the reproductive health needs of violated women and has appropriate validity and reliability. The results of the assessment using the aforementioned instruments can be of great help in promoting the reproductive health of women subjected to violence.

## Plain English Summary

Violence against women is a significant public health problem, as well as is a fundamental violation of women’s human rights. Violence can negatively affect women’s physical, mental, sexual and reproductive health. So there is obviously a need to measure this issue to provide necessary services for women experiencing violence. In current work an appropriate instrument was developed on the basis of the qualitative findings and a literature review. For this purpose, the inductive-deductive method was used to design the expressions and items of the tool. Both qualitative and quantitative methods were used to determine face and content validity. The construct validity was investigated by exploratory factor analysis; in this part of study the sample included three hundred and fifty violated women. The final version of the scale have four factors with total variance of 47.62 and 39 items which are evaluated by Likert scale. The current tool was valid and reliable to measure the reproductive health needs in domestic violated women.

## Résumé

**Introduction:**

La violence est un problème de santé dans le monde ainsi qu’une manifestation essentielle de l’inégalité entre les sexes qui a des conséquences nocives sur la santé des femmes. C’est pourquoi, il est indispensable de reconnaître la santé reproductive des femmes victimes de violence et leurs besoins dans ce domaine pour fournir les services de santé dont elles ont besoin. Considérant que l’un des moyens de la détermination de l’état de la santé reproductive est d’utiliser des questionnaires valides dans ce domaine.

**Méthode:**

La présente étude a été effectuée comme une étude de méthode mixte. La première partie de l’étude a été faite qualitativement avec une approche d’analyse du contenu contractuel et dans cette section, on a mené des entretiens profonds non structurés avec 18 femmes maltraitées et 9 spécialistes. Dans l’étape suivante, un pool d’items a été construit et un questionnaire sur les besoins en santé reproductive des femmes victimes de violence familiale (RHNVWS) a été posé en utilisant l’approche Waltz et en passant en revue les textes et les résultats de la section qualitative avec 39 items. Pour l’évaluation psychométrique des outils ci-dessus, la validité et le contenu, l’analyse et la validation des items ont été effectuées à l’aide d’une analyse factorielle.

**Résultats:**

Selon des résultats de l’analyse factorielle, quatre facteurs avec une variance totale de 47, 62 ont été extraits s’agissant de la « participation des hommes », « les soins personnels », « les services de soutient et de santé » et « les relations sexuelles et conjugales ». Le degré de la cohérence interne de l’outil a été calculé pour de différentes structures de α =. /70 à α =. /89 et pour l’ensemble de l’outil α=. /94. De plus, le coefficient de corrélation intra-cluster pour les structures d’outil était de ICC=. /96 à ICC=. / 99 et pour l’ensemble de l’outil ICC=. /98.

**Conclusion:**

Selon les résultats de cette étude, le questionnaire sur les besoins en santé reproductive des femmes maltraitées, est un outil qui évalue spécifiquement les besoins en santé reproductive de ces femmes et a une validité et une fiabilité appropriées. Les résultats des mesures avec les outils ci-dessus peuvent être utiles à promouvoir la santé reproductive des femmes maltraitées.

## Background

Intimate partner violence (IPV) is defined as a pattern of behavior in a close relationship which leads to some psychological, physical and sexual damage to those who are in the relationship [1]. Violence against women, including IPV which is pervasive universally results to remarkable mental and physical health problems [2]. According to a report which published by World Health Organization (WHO) in 2018, lifetime prevalence of physical and/or sexual IPV among ever-married/partnered women that age between 15 to 49 years was 35% and 31% in Southern Asia and Iran, respectively [3].

The majority of women in South Asian including Iran, who experience IPV tend to hide it due to the following reasons: concerns about their safety and that of their children, fear of family and social rejection, economic problems, fear of losing respect and social status, fear of not being believed by society, as well as emotional dependence on and love of the spouse [4–6].

Regardless of geographical location, color, gender, and culture, violence is a global health problem [7–12] and one of the main manifestations of gender inequality. It is a major obstacle to the attainment of international reproductive health goals since it is directly related to women's access to health care [13]. Health problems are reported more frequently in violated women, compared to other women, and IPV is associated with a range of health issues [7].

Bullying, which is a part of IPV, may reduce women's ability to seek any form of health care [14]. In their study, Akyüz et al. (2008) reported that women with experience of partner violence had more difficulty accessing pelvic exams, compared to other women, since they could not refer to health facilities without their husbands' permission [15]. A similar study demonstrated that the number of beyond facility deliveries without skilled obstetric care was higher among violated women [16]. Along the same lines, the results of a study conducted by Moore et al. (2010) in the United States demonstrated that 74% of violated women reported that their fertility was controlled by their husbands [17].

The results of a study performed by Ludermir et al. (2010) on 1,045 women in northern Brazil pointed out that that the most common type of IPV was psychological violence (25.4%). Moreover, it was found that psychological violence during pregnancy was positively and significantly associated with postpartum depression [18]. In the same context, Sudha et al. (2011) assessed 9,636 Indian married women and denoted that married women's experience of emotional, sexual, and physical IPV in the past 12 months was significantly correlated with the symptoms of sexually transmitted infections. Moreover, the experience of physical violence and acceptance of spousal physical violence was significantly associated with a lower odds ratio for care-seeking [13]. The identification of the relationship between IPV and reproductive health is of paramount importance for health promotion. In this regard, one of the primary healthcare services should be the empowerment of violated women to seek reproductive health care [11, 13]. The impact of violence on health is widely recognized and the results of various studies pointed to the negative effect of violence on women's general health and reproductive health. Nonetheless, a few measures have been taken to shed light on the effect of violence on women's ability to achieve their health needs and health promotion [19, 20].

It is essential to understand the reproductive health status of violated women and the health needs of these women to provide the required services, and one of the ways to promote this awareness is the use of valid questionnaires. Therefore, this mixed-method study aimed to determine the components of reproductive health in women subjected to violence and design a valid and reliable assessment tool.

## Methods

The present study is the second stage of a mixed-method study.

### First phase

In the first part, a qualitative study was conducted using in-depth interviews (IDI) and observation methods. This study was based on conventional content analysis to explain the reproductive health needs of women with experiences of domestic violence. Participants in this section included 18 women within the age range of 20–49 years living in Tehran who were recognized as violated according to the Domestic Violence Questionnaire developed by the World Health Organization.

The inclusion criteria about women were as follows: (1) age range of 20–49 years, (2) absence of any physical or mental illnesses, (3) no history of substance abuse, (4) absence of Acquired Immune Deficiency Syndrome (AIDS) and hepatitis. The exclusion criterion was unwillingness of participants for continue the interview. Moreover, nine key informants in the field of reproductive health, social health, and violence, who have experience of working in the violence service centers and were familiar with the needs of abused women by mean work experience of about 21 years, were also interviewed. The participants including violated women and experts were selected by purposive sampling method which lasted for 9 months. Purposeful sampling is a technique that widely used in qualitative researches for the identification and selection of information-rich cases [21].

Sampling was performed in healthcare and forensic medicine centers in Tehran. The interviews continued until to achieving to the point of saturation which led to the selection of 18 domestically abused women and nine experts in the field.

The data were collected using face-to-face interview/IDI (by unstructured questionnaire) and observation techniques. The interviews were started with an open question then followed by asking exploratory questions. The interview sessions lasted for 35–75 min. For analyzing of interviews, the conventional content analysis method which suggested by Graneheim and Lundman were employed [22]:

1. The recorded data were transcribed verbatim after 2–3 times of listening, and typed.
2. Similar statements were considered as semantic units.
3. The semantic units were summarized and the codes were extracted.
4. The codes were categorized according to their differences or similarities.
5. Finally, considering the hidden concepts, the themes were formed.’

To increase the validity of the data according to proposed criteria by Guba and Lincoln, the researcher maintained a long presence with the participants and devoted sufficient time to collect the data. During long time researcher repeated observation to better understanding of the context of participant views and their experiences (thick description) and trustworthy relationship have been formed among interviewers and participants. In practice, prolonged engagement in the field has no set duration, but ethnographers, agree to spend 4 months to a year [23]

In addition, the presence of the research team and the close cooperation of three experts in the analysis and interpretation of data played an important role in improved validity of the information. Checking was performed in two ways, namely member check and peer check, by other members of the research team. In addition, we used a combination of data collection methods, such as face-to-face interview/IDI (using unstructured questionnaire) and observation techniques [24]. Apart from the research team, an external observer and *code–recode* method were used during data analysis to increase reliability or stability. Simultaneously with the interview, the qualitative content analysis was performed based on the Graneheim and Lundman method [22]using Max Kyoda software 2010.

### Second phase

The inductive-deductive method was used to design the expressions and items of the tool. The items were formed based on the codes extracted from the first phase of the study.

It was simultaneously attempted to find another set of phrases related to this concept through an extensive review of related texts and tools, and finally, the item pool was formed. The instrument was re-examined by the research team, and similar items were removed. Face and content validity, item analysis, and construct validity were performed using exploratory factor analysis for psychometric assessment of the above instrument.

### Face validity

In the present study, in order to evaluate the qualitative face validity, face-to-face interviews were conducted with 10 members of the target group, observing maximum diversity in terms of education, age, and violence severity. The difficulty, relevance, and ambiguity of the items were assessed, and the required modifications were made. In the next step, to eliminate or reduce some disproportionate items and determine the degree of importance of each phrase, the impact score of the item for each of the phrases was calculated based on a five-point Likert scale with the participation of 10 participants. Finally, the items with an impact score of ≥ 1.5 were deemed appropriate, and the rest of the items were removed [25].

### Content validity

Both qualitative and quantitative methods were used to determine content validity. For the assessment of the qualitative content validity, 10 experts and faculty members were asked to provide their corrective feedbacks on the following criteria: clarity and simplicity, grammar, wording, item allocation, and scaling. Content validity ratio (CVR) and content validity index (CVI) were calculated for quantitative content analysis.

To determine the CVR, the theoretical and practical definitions of each construct was provided. Moreover, 14 experts were asked to give their opinions about each of the items based on the three-point scale of "Essential", "Useful but not essential", and "Not necessary". In the current study, since 14 experts examined the primary tool, the minimum acceptable value of the CVR in the Lawshe table was regarded as 0.51 [26]. In order to calculate the CVI, 15 experts were asked to examine each item based on a four-point Likert scale; thereafter, the CVI was calculated. Items with a score greater than 0.79 were deemed appropriate and retained, items with a score within 0.7–0.79 were corrected and revised, and items with a score less than 0.7 were removed [27].

### Content validity index and compliance with the chance agreement

The modified kappa formula was also used to eliminate the probability of chance agreement. Kappa coefficients between 0.4–0.59, 0.6–0.74, and > 0.74 were considered weak, good, and excellent, respectively [28].

### Item analysis

Two methods of determining discrimination index and the loop method were used for item analysis. In the evaluation of the discrimination index, if the correlation coefficient between the item and the whole questionnaire was less than 0.3, the item was removed. In addition, if the correlation coefficient between the two items was more than 0.7, one of those items was removed. In the loop method, the reliability coefficient of all items was initially calculated. If deleting an item reduces reliability, it signifies that this item is well-coordinated with others; therefore, it is a good item [28]. At this stage, before construct validity, the questionnaire was provided to 50 women subjected to domestic violence who met the inclusion criteria, and the above coefficients were calculated for each item.

### Construct validity

The construct validity was investigated by exploratory factor analysis (principal axis factoring and maximum likelihood factor analysis) using Varimax rotation. To select appropriate variables for factor analysis, correlation matrix, Kaiser–Meyer–olkin Measure of Sampling Adequacy (KMO) tests and Bartlett's test of sphericity were used. Eigenvalue and Scree plot were also used to determine the optimal number of factors.

### Reliability

#### Internal consistency

To determine internal consistency, Cronbach's alpha coefficient of items was determined. In addition, the total score and the score of each factor were separately calculated. Items with an alpha of ≥ 0.7 remained in the instrument [29].

#### Stability

In the current study, the test–retest method was used to evaluate stability. In this method, the tool was provided to 50 violated women who met the inclusion criteria in two stages with a two-week interval. Thereafter, inter-class correlation analysis was performed between the scores of the two tests. The coefficients of < 0.5/,0.5–0.75, and > 0.75 were considered weak, moderate, and good, respectively [30].

#### Sample

The sample size was determined based on the number of questionnaire items. As a general rule, the number of samples should be about 4 or 5 times the number of variables [31]. In the current study, the number of samples for exploratory factor analysis was obtained at 6 per item (i.e., 342 samples). Finally, 350 self-reporting questionnaires were provided to participants who were selected by convenience sampling. The obtained data were analyzed in SPSS software (version 22).

## Results

The themes, classes, and subclasses extracted from the qualitative phase of the study are displayed in Table 1. In the next step, 116 items were extracted from qualitative interviews and also according to the review of related studies based on the concept of reproductive health needs of abused women, and the item pool was formed. After three revision sessions held by the research team, some items were merged or deleted, and some others were reviewed and edited. Finally, the number of items was reduced from 116 to 99 items, and a psychometric assessment was performed.

**Table 1:** Main themes and their categories obtained from the data

Main themes	Categories
Need to motivate self-care	Need to improve the sense of self-efficacy in health
	Need to accept responsibility toward health
Need to empower women against violence	Need to optimally design formal and informal support services
	Need to improve compliance with violence
Need to have safe sex life	Need to recognize the female role in sexual intercourse
	Need to build culture to improve the role of women in sexual relations
Need to improve the capacity of reproductive health system in support of domestically abused women	Need to a simple access to economic maternity and safe motherhood services
	Need to have accountable healthcare system to provide abused women with healthcare services
Need to train men to involve in reproductive health issues	Need to improve the attitude and knowledge of men about reproductive health
	Need for men’s emotional investment during pregnancy

### Face validity

Three items were revised in the assessment of face validity in the qualitative stage. The lowest impact factor was obtained at 1.8, and the items received a good score at this stage.

### Content validity

At this stage, some items were removed, and some items were modified. For instance, the phrase "I care about suspicious sexual relationships of my husband" was modified to "I care about suspicious sexual relationships of my spouse due to the transmission of sexually transmitted diseases, AIDS and hepatitis." Finally, 83 items remained after merging and removing duplicate items. In the assessment of content validity, the numerical value of CVR was determined based on the Lawshe Table for Minimum Values and the number of evaluators. If this value was less than 0.51, the items were removed, and the primary instrument with 72 items entered the CVI and kappa coefficient stage. Items with a CVI of ≥ 79 were retained, those which scores within 0.7–0.78 were corrected and revised, and the remaining items were removed. Moreover, the items with a kappa coefficient of < 0.74 were removed, and finally, 66 items remained. The CVI of the whole scale for the present tool was calculated at 0.905, which confirms this important index.

### Item analysis

At this stage, the items whose correlation coefficient with the total score was less than 0.3 were deleted or edited. Moreover, in cases where the correlation coefficient between two items was > 0.7, they were merged and the number of items decreased from 66 to 57.

### Construct validity

The demographic characteristics of the participant in the construct validity assessment are depicted in Table 2. The KMO index in the present study was 0.923, and the result of Bartlett's test of sphericity was significant (P < 0.000). To investigate the number of factors that made up the tool, eigenvalue and scree plot were used. The analysis was performed by considering the eigenvalue of > 1, and finally, four factors were account for 47.62% of variance as follows: men's participation: 20.51%, self-care: 13.24%, support and health services: 8.83%, sexual and marital relationships: 5.02% (Table 3).

**Table 2:** Demographic characteristics of the participants (n = 350)

Variable	Mean ± SD
Age	31.70 ± 6.92
Number of children	1.64 ± 0.79
Marital age	20.77 ± 4.24

Occupational status	Frequency (%)
Housewife	63.42
Worker	0.85
Employed	18.85
Self-employed	12.85
Other	4
Education	
Elementary	6.85
Junior high school	15.14
Senior high school	34.57
Academic	43.42

**Table 3:** Factors extracted from the factor analysis

Row	Item	Load factors			
		Men's participation Var: 20.51%	Self-care Var: 13.24%	Support and health services Var: 8.83%	Sexual and marital relationships Var: 5.02%
1	My husband observes his personal hygiene	0.51			
2	My husband continues to have sex during my menstrual period against my will	0.41			
3	My husband cooperates with me in preventing pregnancy	0.57			
4	My husband does not allow me to visit a doctor for treatment and care	0.63			
5	My husband meets the cost of my treatment	0.70			
6	My husband cooperates with me in seeking treatment for sexually transmitted diseases	0.71			
7	My husband takes responsibility for our married life	0.72			
8	My husband accompanies me to visit a counselor	0.43			
9	My husband has sexual relationships with other women	0.48			
10	I agree with my husband on the number of children and the time of pregnancy	0.57			
11	My husband cares about my needs during pregnancy	0.70			
12	My husband helps me with pregnancy care	0.63			
13	I care about the danger signs in pregnancy		0.64		
14	I care about proper nutrition during pregnancy		0.61		
15	During pregnancy, I pay attention to taking the necessary supplements (e.g., multivitamins and iron)		0.57		
16	During pregnancy and after childbirth, I refer to health centers or offices to receive care		0.47		
17	I am aware of the benefits of condoms in preventing sexually transmitted infections		0.57		
18	I care about having regular and timely Pap smears (cervical cancer tests)		0.43		
19	I care about suspicious sexual relationships of my spouse due to the importance of sexually transmitted diseases, AIDS, and hepatitis		0.55		
20	I know the signs and symptoms of sexually transmitted infections		0.67		
21	I am aware of the mode of transmission and methods of preventing HIV and AIDS		0.60		
22	I care about my personal hygiene and health		0.59		
23	I care about my nutrition		0.52		
24	I know different aspects of violence against women		0.49		
25	I am aware of my personal and marital rights		0.55		
26	Telephone counseling is readily available to me when I am being abused by my spouse			0.63	
27	When I am subjected to violence, the health centers and offices pay attention to it			0.62	
28	When I am abused, I can receive the health care I need			0.65	
29	I have the opportunity to discuss my sexual problems with sexual counselors			0.64	
30	I use counseling and psychological services			0.56	
31	In health centers, enough attention is devoted to psychological problems			0.59	
32	I receive sufficient family and social support			0.45	
33	I can share my needs with my husband			0.51	
34	I can build up an emotional relationship with my husband			0.63	
35	I have the skills needed for a successful marriage			0.40	
36	I am satisfied with my sexual relationship with my husband			0.72	
37	I experience orgasm			0.64	
38	My husband does not care about meeting my sexual needs			0.44	
39	My husband makes fun of my sexual needs			0.57	

### Reliability

To calculate the internal consistency and relative stability of each factor, Cronbach's alpha coefficient and the intraclass correlation coefficient (ICC) calculated for 350 samples. Cronbach's alpha coefficient and ICC for the whole instrument were obtained at 0.94 and 0.98 respectively (Table 4).

**Table 4:** Cronbach's alpha coefficient, Intraclass Correlation Coefficient and Standard Error of Measurement

Factors	Cronbach's alpha	ICC	SEM
Men's participation	0.89	0.99	1.006
Self-care	0.89	0.96	1.718
Support and health services	0.79	0.97	0.869
Sexual and marital relationships	0.88	0.96	1.192
Whole instrument	0.94	0.98	3.520

## Discussion

The results of the qualitative section indicated that the important health problems posed to violated women included lack of sensitivity to health, increased high-risk behaviors, ineffective life skills, insufficient family and social support, spouse's disregard for the physical, sexual, and mental health of women, as well as a health system and reproductive health unresponsive to the needs of violated women. These problems result in special reproductive health needs at individual, family, and community levels. It is essential to identify the health needs of violated women in order to promote their general health and fertility and of course this data could help to promote the health of all women. Therefore, in the second part of this study, based on the data obtained from the qualitative section, the Reproductive Health Needs of Domestic Violated Women scale (RHNVWS) was designed.

To design this questionnaire, all stages of psychometrics assessment, including face validity, content validity, item analysis, construct validity, and reliability were thoroughly performed. Based on the results of exploratory factor analysis and using Varimax rotation, four latent factors were extracted that explained 47.62% of the total variance of the reproductive health needs of women subjected to domestic violence. These factors included: men's participation, self-care, support and health services, and sexual and marital relationships, which yielded a 39-item questionnaire.

The items were scored on a five-point Likert scale ranging from always (5) to never (1), and in some items, from very much (5) to very low (1). Cronbach's alpha coefficient for the whole instrument was calculated at 0.94. As evidenced by the results of the present study, the 12-item construct of men's participation had the highest variance (20.51) among the constructs of this tool. In this construct, the factor loading of items ranged from 0.41 to 0.73; therefore, it can be concluded that men play a key role in the promotion of women's reproductive health [32].

The results of a study carried out by Akhavan and Simbar (2016) demonstrated that men's involvement in reproductive health brings about the following consequences: raising awareness and information transfer to their wives, improving the coverage of family planning services, investing in prevention and control of sexually transmitted infections, increasing men's involvement in pregnancy issues, including early initiation of prenatal care and adoption of appropriate health behaviors [33]. These results largely overlap with the items of male participation in the present questionnaire and highlight the importance of assessing the needs of this area of reproductive health.

Self-care is a key strategy for health promotion and disease prevention [34]. In the RHNVWS, the 13-item construct of self-care accounted for 13.24% of the variance. In this construct, the factor loading of items varies from 0.43 to 0.64 and examines important aspects of self-care in the reproductive health of violated women.

Another construct in the present instrument was support and health services. This 7-item construct accounts for 8.83% of the total variance. The factor loading of the items varies from 0.45 to 0.65, and the items include the support and services that violated women receive from their families, communities, and health centers. The results of a study conducted by Khani et al. (2017) pointed out that the health systems that are unresponsive to sanitary needs and the non-response of the behavioral counseling centers to violence against women are among the important sexual and reproductive health needs of women [35].

According to the World Health Organization, satisfying and safe sex life is one of the aspects of access to sexual rights [36]. The fourth 7-item construct of the present questionnaire is related to sexual and marital relationships which accounts for 5.02% of the variance of the instrument and assesses the various dimensions of sexual and marital relationships. In this construct, the factor loading of the items varies from 0.72 to 0.40.

The Centers for Disease Control and Prevention (CDC) has developed a tool to assess the reproductive health needs of conflict-affected women based on WHO and CDC studies. This instrument encompasses 10 sections, namely demographic characteristics, safe motherhood, family planning, marriage and marital life, sexual history, sexually transmitted infections, knowledge, beliefs and attitudes about Human Immunodeficiency Virus (HIV) and AIDS, gender-based violence, female genital mutilation, and mental health [37].

Another questionnaire similar to the one used in the current study is the Reproductive Health Needs Assessment Questionnaire, developed in consultation with the International Organization for Migration and the United Nations Population Fund. The mentioned questionnaire consists of 114 items in the following sections: demographic information, safe motherhood, family planning, history and practice of sexual activity, sexually transmitted infections, HIV and AIDS, and gender-based violence [38].

The construct of self-care in the present study included items related to family planning, safe motherhood, sexually transmitted infections, HIV and AIDS which were assessed in separate sections in the two mentioned questionnaires. In addition, the sections on marriage, marital life, and sexual history in the two questionnaires were in line with the construct of sexual and marital relationships in the present questionnaire. The two aforementioned instruments have been prepared based on a review of various studies, and their psychometric assessment was limited to content validity. A large number of items and the method of recording the answers in the mentioned questionnaires made them time-consuming for both the respondent and researcher.

The instrument in our study was exclusively designed for conflict-affected women, and its validity has been confirmed by examining the face validity, content, and constructs validity. Moreover, reliability was validated by Cronbach's alpha method. In addition, apart from using transcriptions and opinions of key participants, the items were extracted from interviews with violated women. The present researcher designed this questionnaire to represent reality from the participants' perspectives since the subjects of the present study are a certain group of women who have different experiences.

In the present study, it was attempted to design a valid questionnaire with minimum possible items. Therefore, these scales assess the reproductive health needs of women subjected to domestic violence in four important areas; moreover, it can be applied in a short time due to ease of answering using the Likert scale. The limitation in the first phase of the current study, was difficulty of attracting women subjected to violence to participate in the interview due to the sensitivity of the topic and the feeling of shame also only legally married women were included in this study. In the second phase, regarding to this fact that the primary questionnaire were completed by participants, we trust to their honest answers. One of the strengths of the current study is that the needs assessment was based on interviews with two target groups, including women subjected to IPV and key informants. The consideration of this critical issue in needs assessment increases the reliability of the results.

The results of the current study were confirmed in terms of face validity, content validity, construct validity, and reliability. Therefore, this instrument can be used as a valid and reliable tool for the assessment of the reproductive health needs of women with experience of domestic violence.

## Conclusion

Public health improvement depends on the promotion of women's health. The violated women as a vulnerable group experience more serious health problems and have different health needs, compared to other women. The modification of reproductive health services of violated women and raising the awareness of women and men on reproductive health programs require the assessment of target group needs. In the present study, the use of experts and key informants' opinions and the experiences of violated women through in-depth and qualitative research resulted in the development of the RHNVWS questionnaire with such important features as appropriate reliability and validity, simplicity, and time-effectiveness. Therefore, the present questionnaire, as a practical tool to assess the reproductive health needs of women with experience of domestic violence, can be of great help for health planning to provide the required services to the target group.

## Data Availability

Not applicable.

## Appendix

See Table 5.

**Table 5:** Reproductive Health Needs of Domestic Violated Women scale (RHNVWS)

Row	First sector: men's participation	Always	Often	Sometimes	Rarely	Never
1	My husband observes his personal hygiene					
2	My husband continues to have sex during my menstrual period against my will					
3	My husband cooperates with me in preventing pregnancy					
4	My husband does not allow me to visit a doctor for treatment and care					
5	My husband meets the cost of my treatment					
6	My husband cooperates with me in seeking treatment for sexually transmitted diseases					
7	My husband accompanies me to visit a counselor					
8	My husband has sexual relationships with other women					
9	My husband helps me with pregnancy care					
10	My husband cares about my needs during pregnancy					
		Very much	Much	Medium	Low	Very low
11	My husband takes responsibility for our married life					
12	I agree with my husband on the number of children and the time of pregnancy					
	Second sector: self-care	Very much	Much	Medium	Low	Very low
13	I care about the danger signs in pregnancy					
14	I care about proper nutrition during pregnancy					
15	I am aware of the benefits of condoms in preventing sexually transmitted infections					
16	I care about having regular and timely Pap smears (cervical cancer tests)					
17	I care about suspicious sexual relationships of my spouse due to the importance of sexually transmitted diseases, AIDS, and hepatitis					
18	I know the signs and symptoms of sexually transmitted infections					
19	I am aware of the mode of transmission and methods of preventing HIV and AIDS					
20	I care about my personal hygiene and health					
21	I care about my nutrition					
22	I know different aspects of violence against women					
23	I am aware of my personal and marital rights					
		Always	Often	Sometimes	Rarely	Never
24	During pregnancy, I pay attention to taking the necessary supplements (e.g., multivitamins and iron)					
25	During pregnancy and after childbirth, I refer to health centers or offices to receive care					
	Third sector: support and health services	Always	Often	Sometimes	Rarely	Never
26	Telephone counseling is readily available to me when I am being abused by my spouse					
27	When I am subjected to violence, the health centers and offices pay attention to it					
28	When I am abused, I can receive the health care I need					
29	I have the opportunity to discuss my sexual problems with sexual counselors					
30	I use counseling and psychological services					
31	In health centers, enough attention is devoted to psychological problems					
32	I receive sufficient family and social support					
	Fourth sector: sexual and marital relationships	Always	Often	Sometimes	Rarely	Never
33	I can share my needs with my husband					
34	I can build up an emotional relationship with my husband					
35	I experience orgasm					
36	My husband does not care about meeting my sexual needs					
37	My husband makes fun of my sexual needs					
		Very much	Much	Medium	Low	Very low
38	I have the skills needed for a successful marriage					
39	I am satisfied with my sexual relationship with my husband					

## Abbreviations

IPV: Intimate Partner Violence
RHNVWS: Reproductive Health Needs of Domestic Violated Women Scale
WHO: World Health Organization
CDC: Centers for Disease Control and Prevention
HIV: Human Immunodeficiency Virus
AIDS: Acquired Immune Deficiency Syndrome
IDI: In-Depth Interviews
CVI: Content Validity Index
CVR: Content Validity Ratio
KMO: Kaiser–Meyer–olkin
